# Daratumumab-induced acute angle closure glaucoma: bone marrow transplantation as a possible risk factor and atropinization as a potential solution

**DOI:** 10.1186/s12886-025-04214-5

**Published:** 2025-07-01

**Authors:** Cansu Yuksel Elgin, Ahmet Fırat Atseven, Ofeliya Mammadzada, Özcan Ocakoğlu

**Affiliations:** Istanbul University-Cerrahpasa, Istanbul, Türkiye

**Keywords:** Case report, Acute angle closure glaucoma, Bona marrow transplantation, Atropinization

## Abstract

This case report describes a 29-year-old female with recurrent T-cell Acute Lymphoblastic Leukemia who developed acute angle closure glaucoma (AACG) following daratumumab infusion. The patient, with a history of bone marrow transplantation and head-neck radiotherapy, experienced sudden eye pain and blurred vision minutes after treatment initiation. Ophthalmological examination revealed bilateral closed angles and elevated intraocular pressure. Ultrasound biomicroscopy showed ciliary malrotation and effusion, suggesting choroidal effusion secondary to daratumumab. The condition was successfully managed with topical medications and subsequent infusions were administered with atropine premedication, preventing recurrence. This case highlights bone marrow transplantation as a potential risk factor for daratumumab-induced AACG and demonstrates the effectiveness of atropinization in managing this complication.

## Introduction

Daratumumab, a human anti-CD38 monoclonal antibody, was introduced in 2015 primarily for multiple myeloma and other hematologic malignancies [1]. By 2018, glaucoma-related ocular side effects of daratumumab had begun to be reported [2]. To date, few cases of drug-induced transient myopia and secondary angle closure glaucoma developing on the basis of ciliochoroidal effusion following daratumumab infusion have been documented [2, 3]. 

The acute angle closure glaucoma (AACG) without pupillary block mechanism seen previously with sulfonamide drugs has prompted various hypotheses about the conditions under which daratumumab might cause this effect [4]. Given the frequent occurrence of infusion-related reactions (IRRs) with daratumumab, it is thought that the ciliochoroidal effusion could be another IRR, similar to rhinorrhea, cough, angioedema, or bronchospasm. Standard pretreatment to mitigate IRRs includes intravenous corticosteroids, acetaminophen, montelukast, and diphenhydramine, which might be effective in preventing the accumulation of excess fluid within the choroid and ciliary body, preventing glaucoma development. However, cases of glaucoma crisis have been reported even after routine pretreatments for IRRs before daratumumab infusion [3]. 

Bone marrow transplantation (BMT) may also be a risk factor in the development of AACG following daratumumab treatment, as evidenced by examples in the literature [3, 5]. 

## Case report

The work has been reported in line with the SCARE criteria [6]. A 29-year-old female patient with a history of BMT and head-neck radiotherapy, diagnosed with recurrent T-cell Acute Lymphoblastic Leukemia (T-ALL), developed sudden eye pain and blurred vision within minutes after receiving the first dose of a planned 8-dose daratumumab infusion treatment. The patient had started on a daratumumab 16 mg/kg infusion. As part of the standard protocol, she received routine premedication prior to the infusion, including intravenous dexamethasone, acetaminophen, diphenhydramine, and montelukast. Shortly after, she complained of blurred vision that developed within minutes. The daratumumab infusion was paused upon consultation with the hematology specialist and restarted at 150 cc/h after approximately one hour. The patient reported increased blurred vision 30 min later, leading to a reduction of the infusion rate to 100 cc/h. As the blurred vision complaint persisted below 100 cc/h, the infusion was first reduced to 50 cc/h and then stopped. While visual symptoms were the dominant complaint, mild fluctuations in vital signs were also observed during infusion, consistent with the patient’s known clinical instability due to recurrent refractory T-ALL. At that point, the hematology team initially considered the blurred vision to be a systemic infusion-related reaction (IRR), and visual acuity was not formally assessed. However, as the visual complaints did not improve — and even worsened — during infusion adjustment and cessation, an ophthalmology consultation was requested. Routine premedication was applied before infusion.

**Fig. 1:**
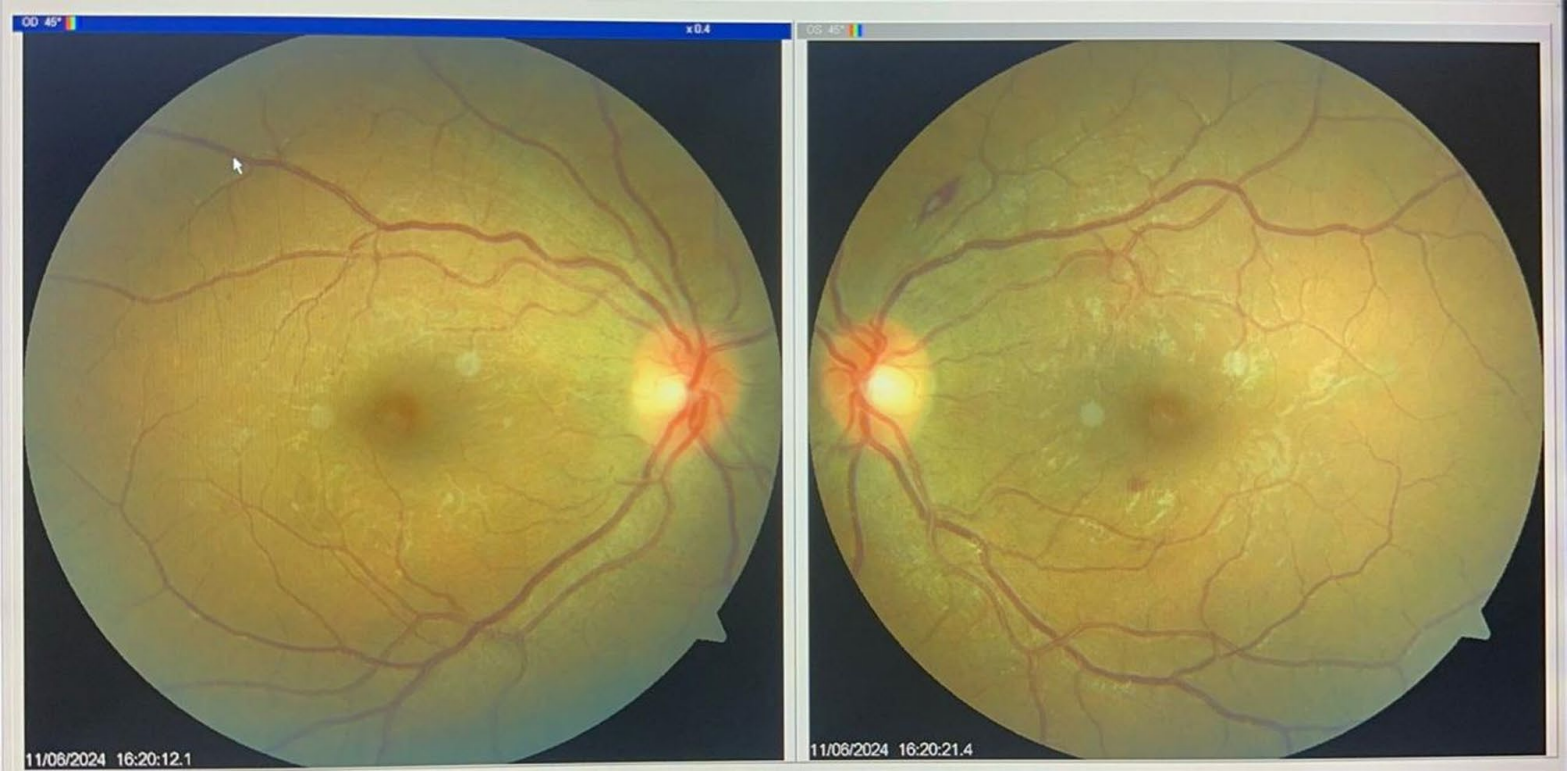
Dilated fundus examination

In consultation, there was no significant ocular history. The best-corrected visual acuity (BCVA) was 5/10, bilaterally. Refraction was OD: -4.00 (15’-1.25) OS: -5.00 (180’-1.75). The slit lamp examination showed no conjunctival injection; however, the corneas exhibited microcystic edema, and the anterior chambers in both eyes appeared shallow. Gonioscopy revealed a closed angle in both eyes, with none of the angular structures visible, classified as 0 according to the Shaffer classification. Applanation tonometry measured intraocular pressure (IOP) at 56 mm Hg OD and 50 mm Hg OS. Dilated fundus examination showed bilateral mild increased vascular tortuosity and Roth’s spot hemorrhages (Fig. 1).

**Fig. 2:**
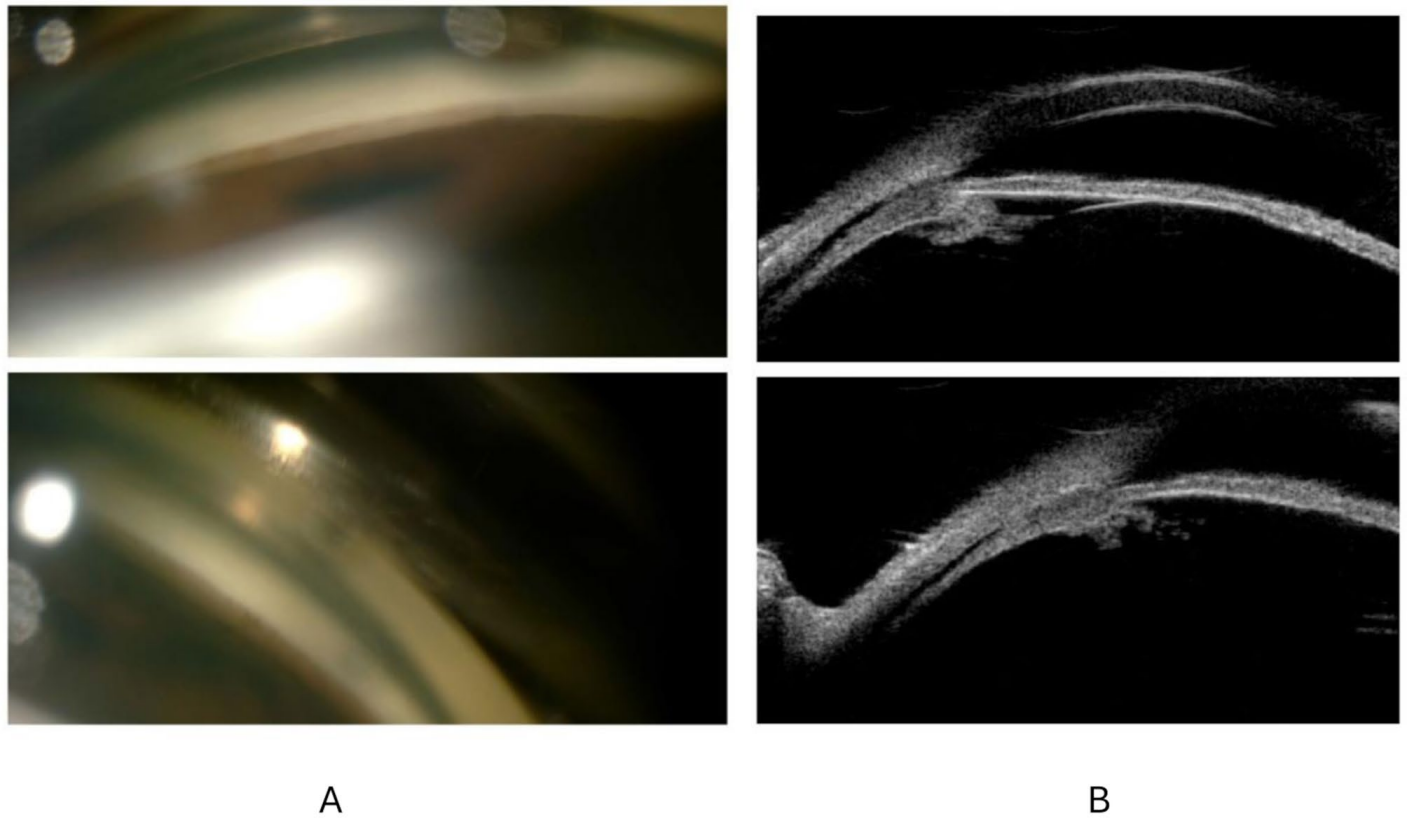
(**a**) Gonioscopic examination (**b**) Ultrasound biomicroscopy

Initial treatment included the administration of tropicamide, dorzolamide-timolol, and brimonidine eye drops, which successfully reduced the IOP to 25 mmHg (OU) within approximately 2 h. During this time, the patient’s symptoms of blurred vision and eye pain also improved. It should be noted that the patient had already received intravenous dexamethasone as part of standard daratumumab premedication, and no additional topical or systemic corticosteroids were prescribed during the ophthalmology consultation. Eight hours later, the patient was transferred from the emergency clinic to the ophthalmology department for advanced imaging and evaluation. After treatment, ocular symptoms subsided, IOP was measured at 22 mm Hg (OU), and BCVA improved to 8/10. Initial evaluation was performed bedside using a handheld slit lamp and rebound tonometry due to the patient’s acute condition. Approximately 2 h after starting treatment, IOP normalized to 25 mmHg, and pain decreased significantly. Imaging tests, including anterior chamber depth measurements, were performed afterward in the outpatient setting. ACD could not be assessed at its most shallow point during the acute phase; the first measurable depths—2.89 mm (OD) and 2.84 mm (OS)—were recorded after symptom resolution. These gradually increased to 3.22 mm (OU) within a week, consistent with the regression of ciliochoroidal effusion. Biomicroscopic examination revealed a regression in anterior chamber shallowing and corneal edema, while gonioscopic examination showed that the angle had opened and was graded as level 3 according to the Schaffer classification (Fig. 2a). No peripheral anterior synechiae (PAS) were noted during initial or follow-up gonioscopic assessments, including with compression. Both Fig. 2a (gonioscopy) and Fig. 2b (UBM) were obtained within 24 h after initial IOP control was achieved with atropine, dorzolamide-timolol, and brinzolamide. Although gonioscopy showed a grade 3 open angle, UBM imaging still demonstrated persistent ciliochoroidal effusion, indicating that anatomical changes may lag behind clinical improvement.

Optical coherence tomography (OCT), fundus photography, axial length, and anterior chamber depth (ACD) measurements were performed. Macular OCT showed no significant pathology, while anterior segment OCT could not be obtained due to technical limitations. Axial length measurements were 22.74 mm (OD) and 22.79 mm (OS), with minimal change over time (final measurements: 22.78–22.80 mm), indicating no clinically significant variation. Ultrasound biomicroscopy (UBM) revealed bilateral ciliary malrotation and effusion, suggesting a diagnosis of choroidal effusion secondary to daratumumab (Fig. 2b).

A follow-up eye examination was conducted 2-weeks later. During this examination, the IOP values were 21 mmHg (OU), the anterior chamber had deepened, fundoscopy findings had regressed, and no pathological findings were observed on UBM. Refractive error regressed to OD: +1.50 (170’-0.75) OS: +1.50 (180’-1.00), and anterior chamber depth changed from 2.89 (OD) and 2.84 (OS) to 3.22 (OU). BCVA increased to full.

The hematologists decided to proceed with the second daratumumab infusion on day 14 in line with the weekly treatment schedule. As prophylaxis, 1% atropine sulfate was initiated 24 h prior to the infusion at a dose of 2 × 1 per day and continued through the infusion day and the following day (total of 3 days). It was then tapered to 1 × 1 per day and discontinued after one week. Both the second and third infusions were administered at the same dose and infusion rate as the first, but no ocular symptoms or IOP elevation occurred under atropinization. OCT and UBM performed 24 h after the second infusion showed a wide angle in both eyes and no suprachoroidal collections. No progression of suprachoroidal fluid was observed on the first day after the second and third infusions (days 14 and 24 after the first infusion). IOPs remained within normal limits (20–22 mmHg), and anti-glaucoma medications were completely discontinued. Following the third infusion, daratumumab treatment was discontinued due to hematological non-responsiveness, as determined by the hematology team.

## Discussion

AACG can occur via multiple mechanisms. The most well-known is pupil block, where pupillary dilation causes the peripheral iris to appose and obstruct the trabecular meshwork. Another important mechanism involves ciliochoroidal effusion, leading to anterior rotation of the ciliary body and forward displacement of the lens–iris diaphragm, as seen in this case.

Additional forms of AACG include lens-induced mechanisms (e.g., phacomorphic glaucoma), vascular causes, and plateau iris configuration, among others. While not all are relevant to this patient’s presentation, it is important to acknowledge the broader range of anatomical and physiological pathways that can result in angle closure.

Daratumumab has been reported to cause not only effusion in the ciliary body but also in the more peripheral choroidal area, leading to a condition known as ciliochoroidal effusion. This may explain the rapid and severe cilioretinal effusion following daratumumab infusions. However, this reaction is rare enough to be reported only as a case report following daratumumab infusions.

Among the cases reported in the literature, two patients underwent bone marrow transplantation [3, 5], with one of them having undergone a second transplantation [5]. This suggests that BMT should be considered a significant risk factor. Similarly, our patient had also undergone a bone marrow transplantation as well as head and neck radiotherapy. This indicates that the disruptions in the body’s barrier systems following these aggressive treatments may allow daratumumab to more easily interact with ocular CD38 expression. Another possibility we considered is that bone marrow transplantation can reshape the immune system and alter inflammatory responses, potentially contributing to these complications.

This case contributes to the literature by highlighting the risk factor of BMT and radiotherapy exposure and demonstrating the effectiveness of atropinization in managing complications. The onset of clinical symptoms within minutes after the first infusion, the striking changes in anterior chamber depth, the pronounced myopic shift, and IOP values exceeding 50 mmHg within hours make it notable. The presumed pathophysiology of AACG in this context involves forward displacement of the lens–iris diaphragm, anterior rotation of the ciliary body, and choroidal effusion, all of which can narrow or close the anterior chamber angle. Cycloplegic agents such as atropine can theoretically counteract this mechanism by relaxing the ciliary body and deepening the anterior chamber. While drug discontinuation is generally preferred in managing drug-induced ocular reactions, in clinical settings where continued administration is necessary—such as in hematologic malignancies—prophylactic atropinization may provide a safe, symptom-preventing alternative. This approach was also supported in a similar case by Saengsirinavin et al., and our case appears to be only the second to demonstrate its clinical utility [7]. Moreover, this report contributes to the literature in two key ways. First, a review of similar cases suggests that bone marrow transplantation—and possibly prior head and neck radiotherapy—may be underrecognized risk factors in daratumumab-associated ciliochoroidal effusion. These observations offer new insight into potential predisposing conditions.

Second, our case demonstrates that prophylactic atropinization may be a viable strategy for preventing recurrence of non-pupillary block AACG in cases where daratumumab therapy must be continued. As such, it offers practical clinical guidance in managing this rare but serious complication.

## Data Availability

All data generated or analysed during this study are included in this published article.
